# Examination of influence of social media education through mobile phones on the change in physical activity and sedentary behavior in pregnant women: a randomized controlled trial

**DOI:** 10.1186/s12905-022-01725-x

**Published:** 2022-05-10

**Authors:** Erfaneh Talebi, Hamideh Mohaddesi, Davoud Vahabzadeh, Javad Rasuli

**Affiliations:** 1 Student Research Committee, Urmia University of Medical Sciences, Urmia, Iran; 2 Non Communicable Disease Research Center, Ilam University of Medical Sciences, Ilam, Iran; 3 Maternal and Childhood Obesity Research Center, Urmia University of Medical Sciences, Urmia, Iran; 4 Maternal and Childhood Obesity Research Center, School of Medicine, Urmia University of Medical Sciences, Urmia, Iran

**Keywords:** Social media education, Mobile phone, Physical activity, Sedentary behavior, Pregnant women, Randomized controlled trial

## Abstract

**Background:**

Nowadays because of some necessities and demands for virtual and remote education, a new model of health approach through mobile-phones is widely used to deal with improving physical activity and its beneficial effect on pregnancy. There are a small number of studies for showing this importance and the efficacy of such methods, so this study was aimed to determine the influence of social networking through mobile phones on changing the physical activity behavior in pregnant women.

**Methods:**

This randomized controlled trial was conducted with parallel groups on 90 pregnant women referring to Urmia health centers in 2018–2019. The participants from various social and economic backgrounds were included. The participants were randomly assigned to a control and a treatment group. Demographic and Pregnancy Physical Activity Questionnaire (PPAQ) questionnaires were filled. In addition to routine cares, the treatment group received educational intervention through social network within 16 training sessions related to physical activity and nutrition in 8 weeks.

**Results:**

The mean weight in both control and treatment groups changed significantly during the study, but in different trends (*P* < 0.001, Mean Difference (MD) = 4.43). At the beginning of the study, control and treatment groups were not different in terms of daily physical activity level (*P* = 0.17, MD = 0.62), meanwhile, there was a statistically significant difference at the end of intervention in the level of daily physical activity (*P* < 0.001, MD = 0.69). Comparison of both groups, based on different categories of activity level at the end of the treatment, showed a non-significant difference in sedentary activities (*P* = 0.89, MD = 0.46), but the intervention led to a significant difference based on the other level of activities; light (*P* < 0.001, MD = 51.94), moderate (*P* < 0.001, MD = 46.87), and sever (*P* = 0.05, MD = 1.07).

**Conclusion:**

Educational intervention based on social networks through mobile phones had an effective role in behavior change in physical activity during pregnancy.

***Trial registration*:**

IRCT20151004024340N15, Registration date05/06/2020.

## Introduction

Because of the change in social and economic patterns in all over the world, the sedentary lifestyle has become a global phenomenon [1]. A sedentary lifestyle is accompanied by overweight, type 2 diabetes, and cardiovascular diseases; therefore, increasing physical activity or reducing sedentary lifestyles is one of the paramount important action for public health [2]. Pregnancy is considered the most sensitive phase in a mother’s life. Having a healthy lifestyle during this period is very important as the fetus is so sensitive and vulnerable to various factors during this time, and the mother’s health has a direct influence on the final fetus health [3].

According to the documents and scientific research, there is a positive relationship between physical activity and the health of pregnant mothers and their children [4]. Physical activity, which includes playing, walking, doing house chores, gardening, dancing, etc., is defined as any kind of body movement that is performed through skeletal muscles. Although such activities consume energy, they should not be confused with sports [5]. American College of Obstetrics and Gynecology suggests physical activity during pregnancy and US instructions recommend healthy pregnant women to practice more than 30 min of physical activity on most days of the week, if there are no any raised side effects for the fetus [6]. Physical activity is an effective way to reduce unpleasant influences of pregnancy such as sleeplessness and feeling tired [7], excessive weight gains of a mother [8], back and pelvic pain [9], constipation [10], high blood pressure [11], depression and stress [12]. Such practices can reduce the severity of pain during labor, improve heart and lung functions, and in the post-labor phase helps the body return to normal quickly [13]. Despite such advantages, the studies demonstrate a significant reduction in the physical activity of women during pregnancy [14, 15]. In this regard, it has been shown that only 23% of pregnant women in America performed the minimum physical activity suggested by the American Congress of Pregnancy and Gynecology [16, 17]. Studies have introduced some factors that they are related with a reduction in physical activity levels in pregnancy, including the lack of training by health care staff about the benefits of safe physical activities and their recommendations in pregnancy [10].

Since the invention of the World Wide Web in 1989, access to the internet and its use has been expanded. Connected devices such as the cell phones and computers and kitchen appliances with internet connectivity have covered most aspects of our lives. Nowadays, people can connect and transfer information without any geographical or time barrier. The common term to describe such information and idea-sharing on the internet or mobile platforms is “social media”, which is defined by the Oxford dictionary as “websites and programs that enable users to create and share contents or to participate in social networks” [18].

Mobile phones are among the important appliances to use social networks, and their extensive access has made them a valuable option for interventions with the purpose of change in behavior [7, 19]. Besides, increasing the availability of health information in a digital format (which can be easily observed and used) and allowing the quick direct interaction between the patient and caregiver are other benefits [20]. During the last decade, in countries with medium income, there has been an increasing research study on mobile health (mHealth). This concept is defined as the use of appliances such as mobile phones or smartphones to transfer health content and various services [5].

It has been reported that the mobile phone is the fastest technology utilized in low and high-income countries. Therefore, a new health approach is increasing use of mHealth nowadays through mobile phones. As a result, attempts to increase the physical activity levels in all populations can be put into practice by applying advanced programs of communication technology [21]. Research by Rothert et al. indicated that weight loss in their study participants has been significantly higher in the group that used the modern technology system compared to the group who just received routine care [22]. But yet there are a small number of studies for showing this importance and the efficacy of such methods. Currently, the importance of training programs depends on its impact on change or the creation of relevant behavior. As mentioned before, physical activities play a key role in improving pregnant women's health, reduction of obesity, pregnancy over-weight, and unbalanced weight gain. Moreover, long and short-term physical activities can have beneficial effects on health during pregnancy and after the labor. Regarding these issues, as well as the influence of maternal health effect on future generation health, the ease of access to the internet for delivering the educational programs through mobile phones for changing the level of physical activity and sedentary behavior in pregnant women, it has been introduced as one of the growing and in progress study era, so this study was designed to examine the influence of social media education through mobile phones on the change of physical activity and sedentary behavior in pregnant women.

## Methods

### Design and sampling

This randomized controlled trial study with parallel groups was conducted on pregnant women referred to Urmia health centers in 2018–2019. The study was last 12 months for execution, from April 2018 to May 2019. This trial was registered in Iranian registry of clinical trials (IRCT) in 05/06/2020 with IRCT20151004024340N15 identifying number. Also this study was verified by the Urmia ethics committee with IR.UMSU.REC.1397.162 code.

### Sample size and sampling method

**Fig. 1:**
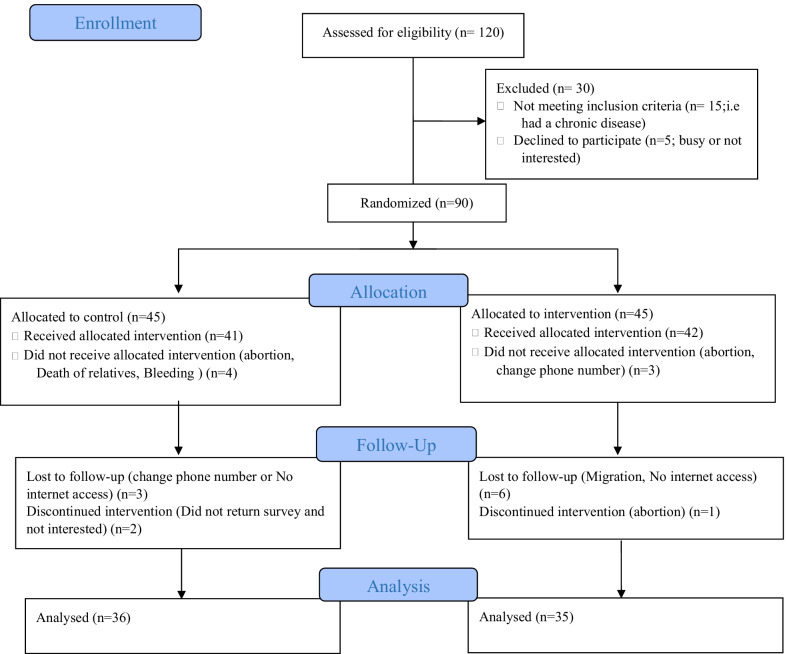
Flow diagram of entering the study and completing of trial by participants

The sample size was calculated according to the Michele Bisson [29] study, because of the similarity in the targeted primary and secondary outcomes, where the moderate and vigorous physical activity (MVPA) index was 11.7 ± 9.5 min/day in their control group and 25.4 ± 20.4 min/day in their treatment group after the intervention. With the 90% of study power (Z_1−β_ = 1.28), and the two-sided alpha level at 0.05 (Z_1−α/2_ = 1.96), and a 10% chance of dropout, the sample size was determined 45 subjects for each group. Therefore, ninety subjects were included in the study. The flow diagram of participants has shown in Fig. 1.

Inclusion criteria in the study were: the tendency to participate in the study, having an ability for reading and writing, be under 20 weeks of gestational age, lack of chronic diseases (i.e., diabetes, hypertension, kidney disease), lack of any medical limitation for improving physical activity by mother, no mental illness and no history of hospitalization (based on the participant's self-report), lack of cerclage and prenatal care, having a smartphone, access to the internet, and residence in Urmia. The exclusion criteria were: having any problems or special diseases in the current pregnancy such as preeclampsia, diabetes, anemia, high-risk pregnancy and cerclage surgery, frequent bleeding during the intervention, premature contractions, reduction of fetal movements, amniotic fluid leakage, diet for a specific disease, and migration from Urmia.

We used stratified sampling method for including subjects from different socioeconomic strata of Urmia. There were three levels of health centers; 1, 2, and 3 according to their social and economic status. Then, two centers from each category were chosen randomly by lottery (drawing). Also participants for each group were randomly assigned with one to one allocation ratio with the block randomization method. First, the blocks were created with the combination of AAABBB by computer and all the possible statuses were identified and an exclusive code was assigned for each. Next, considering the sample size (N = 90) and block sizes (S = 6), 15 blocks were chosen by a simple randomization method by the researcher. Selected subjects were allocated to control or intervention group consecutively by the study manager. All these stages were done under the provision of a consultant epidemiologist and using Random Allocation Software version 1.0.0.

### Data collection tools

In this study, demographic, Pregnancy Physical Activity Questionnaire (PPAQ) questionnaires was used and completed. Height measured with a Stadiometer and weight by a Seca scale. Demographic information questionnaire had questions such as age, weight, height, education level, employment status, economic status, BMI, pregnancy age, number of fetuses, number of pregnancies, history of the underlying disease, history of infertility, cerclage, ectopic pregnancy, and specific disease in the current pregnancy.

Standard PPAQ, which is related to physical activity in pregnancy, includes two parts: Part I consisted of information about individual characteristics and Part II includes 32 questions on physical activities. The questionnaire of pregnancy physical activity consists of 4 groups of questions related to activities: at home (16 questions), community (3 questions), and activity in the workplace (5 questions), and sports and entertainment (8 questions). Within the manual of PPAQ the categorization and classification of daily physical activities based on the intensity and duration of them has been explained well. Based on that manual sum of the values of multiplied intensity and duration for some activities within the questionnaire can predict the amount of MET/Min for each category of activities as sedentary, light, moderate and sever that has pointed in detail in the data analysis section.

After selection of subjects in the first meeting, the researcher introduced herself to mothers and explained the study purpose. Informed written consent was then obtained. Later the questionnaires related to personal information and physical activity were filled in. Body mass index (BMI) was calculated and assessed. The mother’s weight was checked with minimum clothing and without shoes, using a digital scale with a precision of 100 g. People’s height was measured using a wall gauge with an accuracy of 1 cm while standing barefoot by the wall, as the shoulders were in a normal position. BMI was calculated by dividing weight by height squared.

Based on weight status (i.e., normal, overweight, and obese) and through the permuted block randomization, all the pregnant women were randomly assigned to the control and treatment group by Random Allocation Software version 1.0.0. Both groups received individual diets according to BMI, by a nutritionist, who also provided the necessary explanations. All educational content in the format of text, audio, and video files was delivered and provided through of what's app as social media platform. All intervention and control subjects received their specific educational contents according to the prepared study protocol for each group.

### Intervention content

The control group subjects were quiet match and nearly similar in properties with the intervention one. They received a routine pregnancy care that was provided by health care centers and were followed alongside with the intervention group in the same time period. Subjects joined a virtual group in a social network (What's app) that was accessible through both mobile, laptop or PC devices for all subjects from each centers in control group. They received prepared individually diets and materials about that how to track its effect on their weight gain during pregnancy. In parallel, the treatment group also joined a virtual training group in a social network (What's app), that was similarly accessible through mobile, laptop or PC devices for all subjects from each centers in intervention group. Just the difference that was for this group, they received special and prepared educational content as written, audio or video materials to improve their physical activity along with an individually designed diet during 16 sessions in 8 weeks. The content delivered to the virtual group twice a week and in a specified time period. Each session was about 90 min that all educational programs were delivered by an educated and specialized midwife. During different session subjects regarding the advantages of executing appropriate exercises and physical activities during pregnancy, introducing suitable exercises for pregnant women and the manner of their execution was educated. Also in some sessions topics about the necessary precautions and probably health risks with some activities was discussed. Reminder massages was sent twice a week regarding the importance of doing proposed exercises and activities truly, the importance of good adhering to the diet, and announcement about the time of next session and its content. All women’s questions were answered both within the group and in private chat based on the type of question. The respondent rate for both groups was %87.8 in average. Mothers’ weights were recorded after a face-to-face meeting in the fourth week. All delivered massages deposited in virtual group by the end of intervention. Our primary outcome was measuring the amount of changes in the mean daily total physical activity level, while secondary outcomes were measuring the level of changes in other subgroups of daily physical activity alongside with the level of weight gain during pregnancy.

At the end of the intervention, pregnant mothers in both groups filled the PPAQ questionnaires for the second time. The data acquired in this phase were compared with the first one.

### Data analysis

Quantitative data were reported as mean ± standard deviation and qualitative data were expressed in the form of frequencies and percentages. Activity intensity was measured based on the amount of Metabolic Equivalent of Task (MET). To calculate the total MET/Min of activities, the MET level regarding the intensity of activities was multiplied by the amount of time spent per day. The level of daily activity determined based on the amount of MET/Min obtained for activities and their average per day. The categorization for the level of physical activity was done according to the PPAQ specific manual. An activity with MET/Min less than 1.5 was considered as inactivity or sedentary, with MET/Min 1.5–3 as light activity, with MET/Min 3–6 as moderate activity, and with MET/Min more than 6 as a sever activity. An independent t-test was used to compare the data about the groups for comparing them at start and at the end of intervention. A paired t-test was used for comparing changes within groups and comparing before and after data. Also, the chi-square test was used to compare data regard the qualitative variables between the two groups at start of the study. To control the possible influence of confounding factors and better determination of treatment effect, multivariate analysis was done. The significance level for all statistical tests was considered less than 0.05. We used per protocol analysis for comparing the extracted results between study groups. All analyses were performed using SPSS-21 software.

## Results

In this research, 45 subjects were assigned to the each group (Control and intervention group); however, finally 36 subjects in the control group and 35 subjects in the intervention group were succeeded in completing the study period. Two groups were exactly identical in term of basic properties. The quantitative and qualitative demographic characteristics of the participants in each group are shown in Tables 1 and 2.

**Table 1:** Demographic characteristics of the intervention and control subjects for quantitative variables

Variable	Control	Intervention	*P* value
	Mean ± SD	Mean ± SD	
Age	28.94 ± 5.33	29.46 ± 5.78	0.70
Husband age	33.58 ± 5.38	35.34 ± 6.37	0.21
Gravida	2.00 ± 1.15	1.86 ± 0.85	0.55
Marriage duration	6.30 ± 4.53	7.16 ± 5.99	0.50
gestational age	12.03 ± 5.34	13.97 ± 4.38	0.10
BMI	27.02 ± 4.66	27.17 ± 4.37	0.89

**Table 2:** Demographic characteristics of the intervention and control subjects for qualitative variables

Variable	Subgroups	Control	Intervention	*P* value
Volunteers education level (N %)	Elementary	2 (5.6%)	3 (8.6%)	0.63
	Middle school	5 (13.9%)	5 (14.3%)	
	High school	12 (33.3%)	7 (20%)	
	Collegiate	17 (47.2%)	20 (57.1%)	
Husband education level (N %)	Elementary	3 (8.3%)	4 (11.4%)	0.79
	Middle school	5 (13.9%)	5 (14.3%)	
	High school	12 (33.3%)	8 (22.9%)	
	Collegiate	16 (44.5%)	18 (51.4%)	
Job (N %)	Employed	7 (19.4%)	13 (37.1%)	0.10
	Unemployed	29 (80.6%)	22 (62.9%)	
Economic status (N %)	> Expense	6 (16.7%)	5 (14.3%)	0.96
	Equal to Expense	22 (61.1%)	22 (62.9%)	
	< Expense	8 (22.2%)	8 (22.8%)	

As shown in Table 3, the mean level of physical activity and weight in both groups after the intervention was significantly different from the start phase (*P* < 0.001). At the beginning of the study, the control and intervention groups did not have differences from each other in the term of the physical activity level (*P* = 0.17), while there was a significant difference based on the level of activity at the end of the intervention (*P* < 0.001). Also, significant weight changes were seen in both groups in the post-intervention phase compared with the initial phase of the study (*P* < 0.001), but the comparison of groups based on the weight after the intervention did not show significant difference between groups (*P* = 0.20).

**Table 3:** Comparison of mean and standard deviation of physical activity and weight before and after the intervention in the two groups

Variable		Control	Intervention	MD ± SE	*P* value
		Mean ± SD	Mean ± SD		
Total daily physical activity (MET/Min)	Before Intervention	654.80 ± 564.97	830.25 ± 630.99	175.40 ± 142.05	0.22
	After Intervention	75.75 ± 34.41	175.31 ± 61.54	99.56 ± 11.79	< 0.001
*P* value		< 0.001	< 0.001		
Weight (kg)	Before intervention	72.17 ± 13.63	72.41 ± 13.25	0.23 ± 3.18	0.94
	After intervention	79.00 ± 15.31	74.57 ± 13.43	4.42 ± 3.42	0.20
*P* value		< 0.001	< 0.001		

Another comparison between the groups was based on the intensity of activities performed by participants in both groups. At the beginning of the study, there was no significant difference between the two groups based on the intensity of activity in the four categories of activity; sedentary, light, moderate, and sever. For all activities in both groups, compared to the beginning, the intensity of activities at the end of the intervention showed a significant difference. Comparison of the two groups based on different categories of activity intensity at the end of the intervention showed a non-significant difference in sedentary activity, while the intervention yielded significant differences between groups based on light, moderate, and severe activities (Table 4). So, the intervention can make a significant difference between groups in term of the intensity of activities.

**Table 4:** Comparison of mean and standard deviation of different levels of physical activity before and after the intervention among the groups

Variable		Control	Intervention	MD ± SE	*P* value^1^
		Mean ± SD	Mean ± SD		
Sedentary (total MET/day)	Before	17.78 ± 14.67	20.40 ± 19.09	2.62 ± 4.04	0.52
	After	3.79 ± 2.01	3.85 ± 1.84	*0.06* ± 0.45	0.89
*P* value^2^		< 0.001	< 0.001		
Light total MET/day)	Before	50.33 ± 41.61	63.46 ± 45.42	*13.12* ± 10.33	0.21
	After	5.35 ± 3.17	12.77 ± 4.32	*7.42* ± 0.89	< 0.001
*P* value^2^		< 0.001	< 0.001		
Moderate total MET/day)	Before	24.95 ± 32.04	34.40 ± 36.99	*9.40* ± 8.20	0.25
	After	1.61 ± 2.58	8.31 ± 4.88	*6.69* ± 0.92	< 0.001
*P* value^2^		< 0.001	< 0.001		
Sever total MET/day)	Before	0.72 ± 1.97	1.61 ± 3.69	*0.89* ± 0.62	0.16
	After	0.08 ± 0.29	0.23 ± 0.35	*0.15* ± 0.07	0.05
*P* value^2^		0.01	0.07		

^1^Independant sample t-test

^2^Paired sample t-test

A comparison between groups based on the type of activities (i.e., exercise, daily, and home activities) at the beginning and end of the intervention showed that the two groups did not differ significantly in any types of activities in the beginning phase. While at the end of intervention, the two groups showed significant differences in all abovementioned three types of activities. Intra-group comparison based on activity types indicated a significant difference between the groups at the end of the study; however, the difference was more significant in the treatment group. The amount of changes has been shown in the Table 5.

**Table 5:** Comparison of mean and standard deviation of physical activity types before and after the intervention in the two groups

Variable		Control	Intervention	MD ± SE	*P* value^1^
		Mean ± SD	Mean ± SD		
Exercise	Before	2.48 ± 7.78	5.20 ± 7.38	2.71 ± 1.47	0.07
	After	0.25 ± 0.49	1.87 ± 0.93	1.61 ± 0.17	< 0.001
*P* value^2^		0.01	0. 01		
Home activity	Before	61.76 ± 58.16	76.85 ± 60.69	15.09 ± 114.09	0.29
	After	5.45 ± 3.78	14.40 ± 5.70	8.95 ± 1.15	< 0.001
*P* value^2^		< 0.001	< 0.001		
Occupational	Before	3.72 ± 7.65	6.31 ± 12.45	2.59 ± 2.44	0.29
	After	0.62 ± 1.46	1.74 ± 2.70	1.12 ± 0.51	0.03
*P* value^2^		0. 01	0.01		

^1^Independant sample t-test

^2^Paired sample t-test

In the general multivariate test, the value of F for Pillai statistic was 27.7 (*P* < 0.001) and the value of Partial eta was 0.77. These values indicate a significant mean difference between the studied variables in the control and intervention groups. The results of intergroup effects by variables are given in Table 6.

**Table 6:** Results from multivariate analysis of variances (MANOVA) for simultaneously comparing groups

Variable	*F* value	*P* value	Partial eta squared
Daily physical activity level	71.3	< 0.001	0.50
Sedentary activities	0.02	0.88	00
Light activities	68.28	< 0.001	0.49
Moderate activities	38.99	< 0.001	0.43
Sever activities	20.47	0.05	0.05
Exercise activities	82.79	< 0.001	0.54
Home activities	61.09	< 0.001	0.47
Occupational activities	4.73	0.03	0.06

## Discussion

The current study assessed the impact of virtual network-based training via mobile phones on the change in the physical activity patterns of women during pregnancy. The study revealed the effectiveness of executing a designed training program through of social media on the change in women’s physical activity pattern.

In the post-intervention phase, the mean level of physical activity in the intervention group was significantly different from the control group and was reduced less. The level of physical activity in the treatment group was higher at the end of intervention, while it decreased more in the control group. These findings are consistent with the results of Jane et al. (2018), who conducted an educational intervention to track the changes in physical activity behavior. At the end of intervention, they reported a significant reduction in the inactivity in the training group [23].

The results of the current study showed that although the mean intensity of physical activity decreased in both groups, the mean scores in the treatment group were significantly higher compared to the control group. This means that the reduction in the intensity of physical activity in the treatment group was lower than the control group. This may indicate that women in this group had gained a better understanding about the importance of physical activity during pregnancy and therefore showed a greater tendency to do physical activity in the category of light and medium activity level, plus home activity, and exercise than the control group. Similar results have obtained in a web-based intervention study by Rothert et al. and Wilkinson et al. [22, 24]. In the present research, the decrease in the intensity of physical activity in both groups could be due to the increase in pregnancy age. Moreover, pregnant women at this age of pregnancy face certain limitations due to fetal growth and weight gain during pregnancy. In line with the present project, on week 36 of pregnancy, Szmeja et al. has not found a statistically significant difference between the physical activity level of pregnant women in the control and treatment group, after distant training by educational DVDs during the third trimester [25]. Also, Cohen et al. obtained similar results in their study [26].

The findings of the present study are in line with those of Tehrani et al., who assessed the effect of a modern communication technology-based intervention (i.e., multi-media, internet, and mobile phones) on improving the women's physical activity [27]. They showed that the amount of awareness, attitude, and physical activity increased in the treatment group, and finally they concluded that training via mobile phones can have positive effects on the physical activity level of women.

Gaderpanah et al. concluded that training could lead to an increase in physical activity level or less reduction of the intensity of physical activity. In their study, the mean physical activity intensity increased considerably in the treatment group [28]. Moreover, after the educational intervention, an increase in the intensity of activities was seen. In the present study, the technology-based educational intervention led to the prevention of a sharp decrease in the intensity of physical activity in the intervention group compared to the control group. This difference in the effect of the intervention may be due to the difference in the intervention type. More ever, in their study, the training intervention was performed in the form of counseling method based on 5A Model.

According to the results of the present study, the virtual network-based intervention in the mobile platform can make a significant difference between the two groups in all three categories of activity intensity (i.e., light, moderate, sever), but did not make a significant difference in sedentary activity (*P* = 0.89). These findings are consistent with those of Bisson et al. as in their study technology-based education led to an increase in all three categories of activity including light, moderate, and sever activities [29]. Findings of the present research like many other the previous studies indicated that the mean exercise activity of women is generally low. For instance, in a study from Thailand, most women considered exercise as an effective and advantageous activity during pregnancy and believed that its benefits outweighed its disadvantages; nevertheless, they had little physical activity during pregnancy. The results of their study showed that 40% of pregnant women, without any medical limitations, finalized their pregnancy without exercising [30].

Some studies suggest that the mean of the total activities has not been statistically changed during pregnancy [8, 25, 31]. They examined the effect of educational intervention on women’s physical activity levels (i.e., light, moderate, sever, and sedentary), and their results showed that the use of mobile software to intervene in the field of physical activity during pregnancy does not cause a significant change in the moderate and severe levels of activity in intervention group [32]. These findings are in contrast with our results. The difference in the results can partially be attributed to differences in the target population and differences in the used platform for the intervention, how to implement and follow up the intervention, the duration of the intervention, and the lack of active training in the intervention. Another inconsistent study is the Magann et al. [33] one. Some part of differences might be due to differences in educational content, and how the intervention is implemented, cultural differences, and the resulting changes [33].

Some limitations of our study that can be declared here are the vulnerability of the pregnancy, the lack of access to a smartphone for some people, and the fear of complications in some people during pregnancy. In this study, the possibility of drop out in the sample size was controlled effectively. Despite the positive effect of intervention via the social network in the mobile context on the behavior of women’s physical activity, it was not possible to control and obligate them to perform all suggested physical activities completely, and also their ability to select and continuing their participation in the study affected the results to some extent. However, the reminders through the posted messages in the created social group, phone calls, and frequent follow-ups with short intervals could somehow reduce these effects. One another limitation was the low speed of the internet and in some cases the internet interruption or problems related to filtering of used social network in Iran. Nevertheless, it seems that programming interventions based on the social networks in the mobile context can have a positive effect on the level of physical activity during pregnancy.

One of the strengths for the present study is the frequent and short-interval follow-up and the regular repetition of the educational message in the virtual group. Another one was recruiting a group of relevant specialists (including a nutritionist, physical activity specialist, health specialist, and midwife) for preparing the useful training program for each area. Moreover, the use of alternative social platform (Telegram in some instances) to ensure timely access to educational content and programs was another advantage of the present study.

## Conclusion

Educational intervention based on the social networks in the context of mobile phones has an effective role in changing the behavior of women during pregnancy and can to motivate the pregnant women to modify their behavior for promoting their health during the pregnancy. Therefore it can be recommended that providing the educational and interventional programs during pregnancy, especially based on the eHealth and mHealth in changing women’s behavior in physical activity is one the cost benefit strategies.

## Data Availability

The dataset used in this study is available from the corresponding author on reasonable request.

## Abbreviations

PPAQ: Demographic and Pregnancy Physical Activity Questionnaire
BMI: Body Mass Index
MET: Metabolic Equivalents
UMSU: Urmia Medical Sciences University
